# Internet-Based Intervention to Promote Mental Fitness in Mildly Depressed Adults: Design of a Randomized Controlled Trial

**DOI:** 10.2196/resprot.1791

**Published:** 2012-04-26

**Authors:** Linda Bolier, Merel Haverman, Jeannet Kramer, Brigitte Boon, Filip Smit, Heleen Riper, Ernst Bohlmeijer

**Affiliations:** 1 Trimbos Institute, Netherlands Institute of Mental Health and AddictionInnovation Centre of Mental Health and TechnologyUtrechtNetherlands; 2 University of TwenteDepartment of Psychology, Health and TechnologyEnschedeNetherlands; 3 Trimbos Institute, Netherlands Institute of Mental Health and AddictionDepartment of Public Mental HealthUtrechtNetherlands; 4 VU University Medical CentreDepartment of Epidemiology and BiostatisticsEMGO+ Institute for Health and Health Care ResearchAmsterdamNetherlands; 5 VU UniversityDepartment of Clinical PsychologyAmsterdamNetherlands; 6 GGZ InGeestRegional Mental Health Service CentreVU University Medical CentreAmsterdamNetherlands; 7 Leuphana UniversityLüneburgGermany

**Keywords:** Mental health, public health, preventive medicine, depression, well-being, happiness

## Abstract

**Background:**

Investing in mental well-being is considered a supplement to current mental health service delivery in which the treatment and prevention of mental disorders are core components. It may be possible for people to enhance their well-being by boosting their “mental fitness.”

**Objective:**

Psyfit, an online, multi-component, fully automated self-help intervention, was developed with the aim of improving well-being and reducing depressive symptoms. The efficacy and cost-effectiveness of this intervention will be examined in a randomized controlled trial.

**Methods:**

In this two-armed randomized controlled trial, a total of 290 participants will be assigned to use Psyfit (experimental condition) or to a 6-month waiting list (control condition). Adults with mild to moderate depressive symptoms interested in improving their mental fitness will be recruited from the general population through advertisements on the Internet and in newspapers. Online measurements by self-assessment will be made prior to randomization (pre-test), 2 months after baseline (post-test), and 6 months after baseline (follow-up).

**Results:**

The primary outcome is well-being. Secondary outcomes are depressive symptoms, general health, vitality, and economic costs. Analysis will be conducted in accordance with the intention-to-treat principle.

**Conclusions:**

This study will examine the efficacy and cost-effectiveness of an online intervention that aims to promote well-being in people with elevated levels of depressive symptoms. If shown to be effective, the intervention could prove to be an affordable and widely accessible intervention to improve well-being in the general population.

**Trial Registration:**

The study is registered with the Netherlands Trial Register, part of the Dutch Cochrane Centre (NTR2126).

## Introduction

Depression, anxiety disorders, and alcohol dependency are highly prevalent mental disorders [1,2] and among the 10 disorders with the greatest disease burden [3]. These disorders are associated with reduced quality of life [4] and high economic costs due to productivity losses and high levels of health service uptake [5]. In addition, many more people suffer from poor well-being [6], subclinical depression [7], and stress at work [8] putting them at greater risk of developing a mental disorder later on. In the current dynamic and innovative knowledge- and service-driven economy, more people are faced with mentally demanding jobs. This places unique demands on people’s emotional, social, and cognitive capacities [9,10]. Countries are challenged by the adverse consequences stemming from these demanding circumstances, such as burnout, absenteeism, and subsequent economic costs. Therefore, it is important is to reach people at an early stage when the development of a full-blown mental disorder might still be prevented. So far, preventive interventions developed for this reason focus mainly on the prevention of mental health problems and disorders, and less on the improvement of well-being [11].

The World Health Organization (WHO) defines mental health as “a state of well-being in which the individual realizes his or her own abilities, can cope with the normal stresses of life, can work productively, and is able to make a contribution to his or her community.” This definition of mental health represents a paradigm shift from focusing on the narrow medical constructs of illness and disease only, to embracing well-being as well [12].

The WHO definition also underscores the notion that mental health is not merely the absence of mental illness. Well-being and mental illness, although correlated, are independent concepts and not just opposites on a single continuum. People presenting with low levels of well-being have similarly poor psychosocial outcomes as people suffering from mental illness [13]. Likewise, people with mental disorders are capable of experiencing well-being, to some degree [14]. In general, people with high levels of well-being are physically healthier, live longer, are more productive at work, and use less health care [15,16]. Longitudinal and experimental evidence suggests that positive affect and well-being may generate these desirable outcomes [17]. The available evidence suggests that the enhancement of well-being might be a valuable public health strategy in mental health promotion.

With this positive approach to mental health in mind, we developed an Internet-based self-help intervention (“Psyfit”) aimed at the promotion of well-being. Below, we elaborate on the public health rationale for this type of intervention, including the definition of well-being, the relevance of the Internet as an implementation vehicle, and the use of positive psychology interventions as a starting point.

### Definition of Well-being

There are three different types, or concepts, of well-being. The first is subjective well-being, which is a cognitive and/or affective appraisal of one’s own life as a whole and seeking a balance between positive and negative emotions [18]. The second is the concept of psychological well-being. This concept builds on the work of Carol Ryff who was dissatisfied with the emphasis on subjective well-being and focused more on the optimal functioning of the individual [19]. In Ryff’s view, psychological well-being contains six elements: self-acceptance, autonomy, environmental mastery, personal growth, purpose in life, and personal relations with others. According to this view, the attainment of personal happiness is not the goal in life, but rather self-actualization and meaning. The third concept arose from the work of Corey Keyes who called for a broader and less self-centered orientation towards well-being and for the expansion of subjective and psychological well-being to include social well-being; in other words, a complete state model of mental health [6]. Social well-being refers to the extent to which a person feels at home in society, trusts other people, and makes sense out of the world. The WHO’s definition is clearly rooted in this perspective.

### Using the Internet: eHealth

The promotion of well-being requires the delivery of effective and accessible interventions aimed at sustainable behavioral change. The Internet might offer the opportunity to reach this goal. Using persuasive technology techniques, programs designed to change attitudes and behaviors can be made for computers, game systems, and mobile devices [20] so that they are highly engaging and enjoyable at the same time. Internet interventions are defined as highly structured; self- or semi self-guided; founded on evidence-based, face-to-face interventions; tailored to provide follow-up and feedback; personalized to the user; interactive; and enhanced by animation, audio, or video technology (if possible) [21].

eMental Health interventions can vary from plain information, tailored advice, single exercises, and interactive self-help programs to structured online therapies with or without guidance from a therapist. The major advantages of eMental Health are that Internet interventions can be offered on a broad scale, they are able to engage hard-to-reach people, and they can reduce therapists’ time (ie, reduce costs) [22].

In the field of Internet interventions, the efficacy of interventions aimed at the reduction of depressive symptoms or anxiety has been demonstrated meta-analytically [23]. The majority of these programs are based on cognitive behavioral therapy [24] and problem-solving therapy [25,26].

### Positive Psychology Interventions

The positive psychology movement has developed many interventions that focus on flourishing and positive functioning. These include counting one’s blessings [27-29], practicing kindness [30,31], setting personal goals [32-34], expressing gratitude [28,29], and using personal strengths [28]. A comprehensive meta-analysis of 51 positive psychology interventions has demonstrated moderate effect sizes for enhancing well-being and reducing depressive symptoms [35].

However, experimental research on well-being interventions offered over the Internet is still scarce and the results are mixed. A randomized controlled trial with 2 separate interventions (working with your strengths and problem solving) and a placebo control group showed mixed results [36]. Well-being was improved, but there was not a significant impact on mental illness. In another trial [28], the Internet was used for the recruitment of participants and the collection of data. The single exercises (using signature strengths in a new way and recapitulating three good things) enhanced well-being and reduced depressive symptoms for up to 6 months. Also, “writing and reading a gratitude letter” was effective, but only in the short term. However, a more critical look at the interventions reveals that these exercises were not truly Internet-based interventions as described in the previously cited definition by Ritterband [21] because the interventions were neither interactive nor personalized. Another two randomized controlled trials examined the effects of multi-component interventions [37, 38]. In a workplace setting, an intervention called “Resilience Online” did not demonstrate any significant effects [37]. In another study using an online version of positive psychotherapy, depressive symptoms were significantly reduced, but there was no improvement in subjective well-being [38].

### The Current Study

The aim of this study is to evaluate the efficacy and cost-effectiveness of Psyfit, an online well-being program [39]. The study will add to the existing literature by testing a multi-component and flexible Internet-based intervention to promote well-being. To date, the research in this area is limited to either single interventions focusing on one well-being exercise at a time [28, 36] or to multiple protocol-based interventions [37,38]. In these studies, participants in the intervention groups are allocated to an inflexible intervention, although most people would prefer to choose what they need and feel up to doing [30,40,41]. For this reason, Psyfit offers a choice of different interventions that participants can tailor themselves.

The primary objective of this study is to evaluate the effectiveness of the Psyfit intervention in comparison to a waiting list control group. We hypothesize that the intervention group will demonstrate a significant increase in well-being and a reduction in depressive symptoms at post-test and follow-up compared to the control group. Secondary study objectives are to conduct an economic evaluation and to examine if particular subgroups benefit differently (ie, more or less) than others from the intervention.

## Methods

### Study Design

This study is designed as a randomized trial with two parallel groups. In the experimental condition, participants will receive 2 months free access to Psyfit. In the control condition, participants will be put on a waiting list for 6 months before they are offered access to Psyfit. The study is designed to compare the efficacy and cost-effectiveness of Psyfit relative to the waiting list control condition. A secondary objective is to examine whether certain groups (eg, based on depressive symptoms, gender, and education level) benefit differently from the intervention. Participants in both conditions will have unrestricted access to professional help, if needed. The study protocol, interventions, participant information, and informed consent procedure have been approved by the Dutch Medical Ethics Committee for Mental Health Care (METIGG), under registration number 9218.

### Inclusion and Exclusion Criteria

The participant group is defined as everyone willing to improve their “mental fitness.”

Participants will be included if they: (1) are 21 years or older; (2) present with very mild to moderate depressive symptoms with a score between 10-24 on the Center for Epidemiological Studies Depression Scale (CES-D); (3) have moderate or low levels of well-being as measured with the Mental Health Continuum-Short Form (MHC-SF); (4) have access to a computer and the Internet; and (5) have sufficient knowledge of the Dutch language. The CES-D [42] and MHC-SF [43,44] inclusion and exclusion scores are based on established cut-off points.

People with serious depressive symptoms (CES-D score =>25) or active suicidal thoughts or plans (determined from the Web Screening Questionnaire [45]) will be excluded from this study. Those who fail to meet these selection criteria will be notified by email and will be advised to contact their general practitioner if their depressive symptoms exceed the threshold limit. In cases of suicidal ideation, people will be urgently referred to the national online suicide-prevention platform for help.

### Recruitment

Participants will be recruited through banners on Internet websites related to mental health and well-being. In addition, advertisements will be placed in newspapers and monthly magazines on health-related topics.

The recruitment message for the study is formulated positively (ie, not with a focus on symptoms and problems): “Would you like to increase your mental fitness? Would you like to feel better? Improve your mental fitness and participate in our study of an online self-help program, Psyfit.” The analogy is made with physical fitness: “There are certain lifestyle behaviors you could adopt that can make you feel mentally fit.” Preliminary focus group research has shown that people with minor mental health problems, those experiencing stress, or those who just “don’t feel good” are attracted by the “mental fitness” message [46].

The advertisements will include the website address where people can register for Psyfit (www.psyfit.nl). This website contains complete information about the study and a demonstration video of the intervention. Those interested in participating can leave their name and email address. The email and Internet Protocol (IP) addresses will be checked for multiple registrations. Following this, prospective participants will receive an email with additional information about the study and a link to the online informed consent form and online questionnaire.

### Randomization

The online randomization procedure will be carried out at the individual level. After returning the informed consent form and completing the baseline questionnaire, people who meet the inclusion criteria will be randomly allocated to the experimental group (Psyfit) or to the waiting list and they will be notified by email. Randomization will be stratified by gender, education, and severity of symptoms based on CES-D scores (scores between 10-15 and 16-24). A computer program will allocate participants using a generated randomization list. Block randomization in blocks of two will be performed to ensure equal distribution of participants across conditions.

### Intervention Group: Psyfit

**Figure 1:**
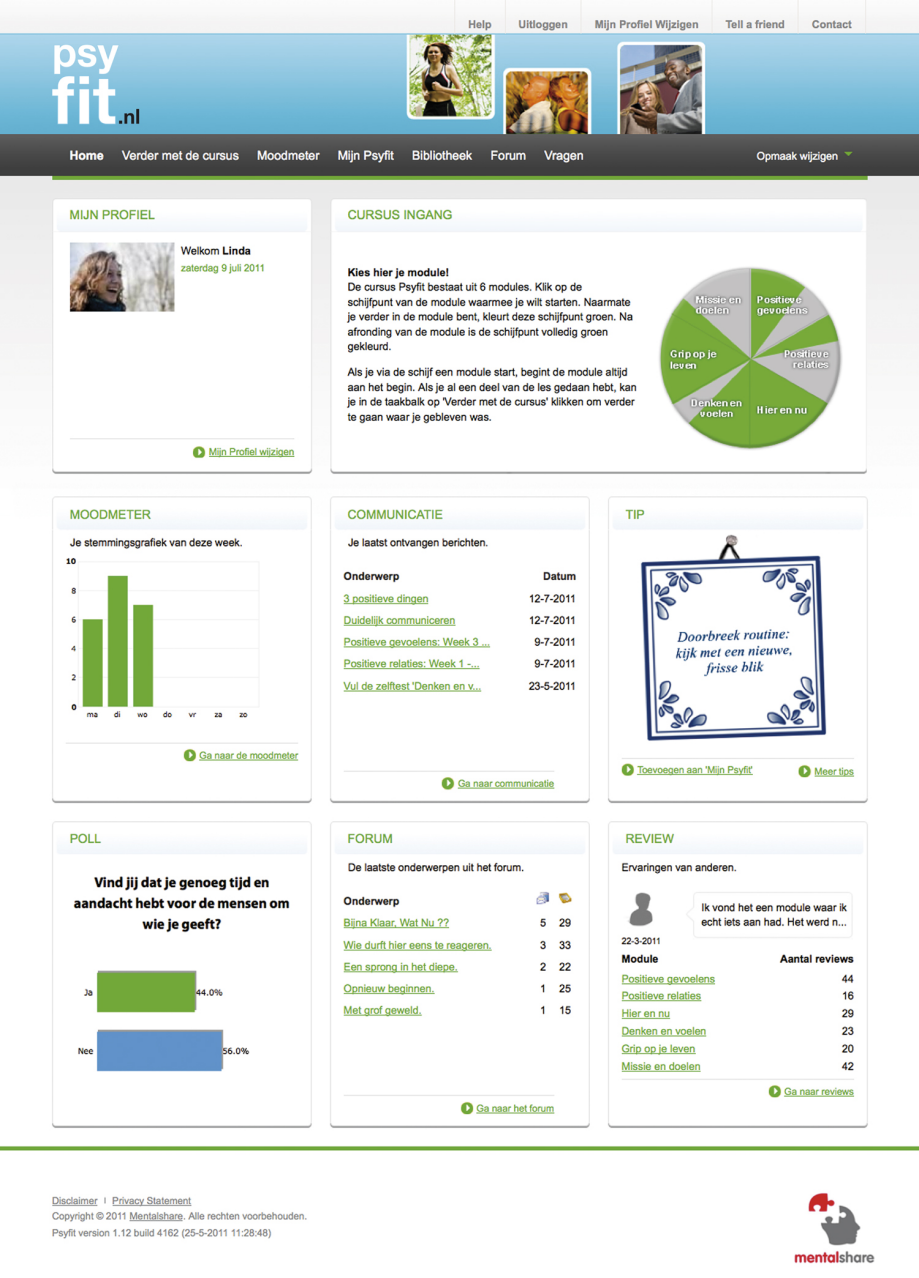
Screenshot of the Psyfit portal.

Psyfit is offered as an online and fully automated self-help intervention without active support from a therapist (see Figure 1 for a screenshot of the intervention). Participants tailor their own intervention program to their personal needs and measure their progress by several self-tests. In addition, they can discuss their experiences in an online community accessed via Psyfit.

The content of the well-being program, Psyfit, is based on an extensive literature review [10]. Elements in the intervention originate from positive psychology [28,35], mindfulness [47], cognitive behavioral therapy [48], and problem-solving therapy [49].

Psyfit consists of six modules, each containing a 4-week program:

1. Mission and goals (living from a deeply felt mission and personal values);

2. Positive feelings (positive thinking and working on your positive affect);

3. Positive relations (connection with other people and your environment);

4. Living in the moment (consciously living and enjoying);

5. Thinking and feeling (change negative thinking patterns, optimistic thinking); and

6. Master your life (managing personal energy, stress, and problems).

In theory, each module is likely to have impact on well-being. During the study, the Psyfit website is accessible only to the participants in the experimental group. An email will be sent to each participant assigned to Psyfit with a personal username and password. From the moment the participant logs on, a 2-month free access to the intervention is activated. If a participant doesn’t log on, a reminder email with the log-on codes will be sent after one week and, if necessary, after two weeks and again after three weeks. Participants are allowed to use the program at any time they want during the trial period. Participants are advised to choose and finish at least one module during the intervention period. During each 4-week module, participants receive background information on the specific subject, view short films, and receive a weekly assignment that they are expected to complete during that week. On average, this takes 20 to 30 minutes each day.

Participants are free to use all other functionalities offered in Psyfit and can always choose to start a new module. For an overview of functionalities, see Textbox 1.

**Functionalities in Psyfit**

- General self-test to assess individual well-being level beforehand and after 2 months.
- A personal plan in which the participant can reflect on his or her goals, motives, and pitfalls.
- A “mood meter” for monitoring changes in the mood of the participant. The outcomes are presented in a graph.
- Automatic email service twice a week with reminders, tips, and advice.
- Online community for sharing experiences and peer-to-peer support.
- Contact form: participants can ask questions and receive feedback from a psychologist via email. If required, the participant is referred to professional care. Technical assistance is also provided.
- “My Psyfit”: the participant can download and print out a personal PDF blueprint of the intervention with all the modules, exercises, and progress measurements completed.
- Videos: each module starts with a video showing a Dutch expert explaining the relevance of this particular module.
- Module self-tests: each module starts and ends with a short self-test to see if the particular skill has improved.

The participant can work through the intervention independently (self-help) but can fill in a contact form with a question if necessary. Psyfit could be likened to a toolbox from which people can “pick and mix” whatever they like and need. The web statistics module will systematically track and trace the actions of each participant, such as the number of log-on times, the time spent on the website, and the modules chosen. This enables adherence to the intervention to be examined.

### Control Group

Participants in the control condition are placed on a waiting list for 6 months. After a 6-month follow-up assessment, they will receive their personal user name and password for Psyfit.

### Assessments

The primary outcome measure is well-being. Secondary outcome measures include depressive symptoms, general health and vitality, and economic costs (as measured by health care use, days of absence, inefficient job performance, and work productivity). For an overview of outcomes and instruments, see Table 1.

Measurements will be taken at baseline (pre-test), directly after the intervention (post-test, 2 months after baseline), and 6 months later (follow-up). All measurements are self-report measures and will be administered via email with a link to the questionnaire on the Internet.

High dropout rates are a characteristic feature of online trials and can be as high as 50% [50]. Therefore, email reminders and rewards (eg, raffle of six iPods and vouchers) for filling in the questionnaires will be used. It is expected that this will lead to a lower attrition rate.

**Table 1:** Questionnaires and assessment times.

		T0	T1	T2
Questionnaire^a^	Measurement	Pre-test	Post-test^b^	Follow-up^c^
MHC-SF	Well-being/positive mental health	X	X	X
WHO-5	Well-being	X	X	X
CES-D	Symptoms of depression	X	X	X
MOS SF-36 subscales	Vitality and general health	X	X	X
TIC-P and PRODISQ	Health service uptake and production losses	X	X	X
CSQ-8	Client satisfaction		X	
Demographics	Age, gender, education, marital status, job status	X		

^a ^all questionnaires are discussed within Instruments section

^b ^2 months after T0

^c ^6 months after T0

### Instruments

#### Well-Being and Positive Mental Health

Well-being will be assessed with two questionnaires measuring different concepts. The Mental Health Continuum-Short Form (MHC-SF) [51] is a 14-item self-report questionnaire that categorizes measures of well-being as “languishing,” “moderate,” or “flourishing.” Participants rate the items on a 6-point scale from 0 (never) to 5 (every day). The MHC-SF measures subjective well-being as well as psychological and social well-being. These aspects are all addressed in Psyfit; therefore, this questionnaire was chosen. The MHC-SF has shown good internal consistency (> .80) and discriminant validity [44,52].

The WHO-Five Well-being Index (WHO-5) contains 5 positively formulated items on mental health [53]. Participants are asked to rate the items using a 6-point scale ranging from 0 (never) to 5 (all of the time). The WHO-5 has been validated in different populations [53] and is useful as a screening instrument for depression [54]. It was chosen as a measurement instrument because of its brevity and the concurrent validity with depression.

#### Depressive Symptoms

Depressive symptoms will be measured by the Dutch version of the Center for Epidemiological Studies Depression Scale, the CES-D [55]. The CES-D is a 20-item self-rating scale with item scores ranging from 0 to 3 (higher scores indicating more depression), and a total score from 0 to 60. The CES-D has acceptable reliability and validity with a cut-off score of 16 for mild depressive symptoms [42] and a cut-off score of 25 for severe depressive symptoms [56]. When applied via the Internet, the CES-D appears to be a reliable and valid instrument [45].

#### Vitality and General Health

Two quality of life related constructs, “vitality” and “general physical health” will be measured with two scales from the Medical Outcomes Study Short Form-36 (MOS SF-36) [57]. The vitality subscale contains 4 items which are rated on a 4-point scale ranging from 1 (all the time) to 6 (never). The “perception of general health” subscale consists of 5 items which are rated on a 5-point Likert scale (1 = “exactly right” to 5 = “exactly wrong”). The MOS SF-36 has demonstrated good reliability and validity [58].

#### Economic Costs

The economic evaluation will be conducted from a societal perspective. It includes utilization costs of any type of health care and medicines (direct medical costs), costs incurred by the participants for travel and parking (direct non-medical costs), and costs due to production losses (indirect non-medical costs) [59,60]. All costs will be expressed in Euro (€) on an annual per capita basis for the reference year 2010. Data on direct medical and non-medical costs are obtained by using the Dutch Cost Questionnaire for Psychiatry (TIC-P) [59]. Six items from the Productivity and Disease Questionnaire (PRODISQ) will be used to measure indirect non-medical costs [61] stemming from productivity losses due to days of absence and presenteeism/inefficient job performance.

#### Other Variables

Participant satisfaction with the intervention will be measured with an adapted Dutch version of the Client Satisfaction Questionnaire-short form (CSQ-8) [62,63]. The internal consistency of this scale in the Dutch population is very high (Cronbach alpha =.93). The 8-item self-report questionnaire has a scale ranging from 1 to 4 and a total score range from 8 to 32.

Furthermore, participants will be asked about important life events and whether they are currently receiving treatment from a mental health specialist.

### Sample Size

Depressive symptoms and well-being are used as a starting point for the power calculation. We aim to be able to show differences between Psyfit and the waiting list control condition with a standardized effect size (Cohen’s *d*) of 0.33 or larger. A standardized effect of 0.33 can be considered as the lower limit of a moderate clinical effect [64] and is based on a meta-analysis of well-being intervention research [35] and a recent randomized controlled trial [36]. To demonstrate this effect, and assuming an alpha of 0.05 and a statistical power (1-Beta) of 80%, we need 145 participants in each condition; therefore, we need 290 participants in total for the trial.

### Analysis

Results will be reported according to the Consolidated Standards of Reporting Trials (CONSORT) statement regarding eHealth [65,66]. We will adhere to the intention-to-treat (ITT) principle, which means all participants who have been randomized will be included in the analyses. Missing data at T1 and T2 will be imputed using the expectation-maximization (EM) method, as implemented in Statistical Package for the Social Sciences (SPSS) Missing Value Analysis. The program imputes missing values by maximum likelihood estimation using the observed data in an iterative process [67]. In online trials dropout is to be expected, and sometimes a large amount of missing data has to be estimated. Although ITT analysis is the approach of choice according to the CONSORT statement, there may also be pitfalls, such as when the missing-at-random assumption is not plausible and the results of the analysis are subsequently biased [68]. Therefore, a completers-only analysis and a per-protocol analysis will be conducted in addition (sensitivity analysis). Reasons for dropout in the study will be checked at random by telephone follow-up (dropout analysis).

To examine differences between the two conditions, we will use multiple regression analyses with the clinical outcomes on continuous measures (MHC-SF, WHO-5, CES-D, and the vitality and general health scales from MOS SF-36) as dependent variables and an intervention-control group dummy as predictor variable. We will compute standardized effect sizes (Cohen’s *d*). Cohen’s *d *is computed by subtracting the mean post-test intervention score from the mean post-test control group score and dividing the difference by the pooled standard deviation [69].

Moderator analyses will be conducted to examine which groups benefit more (or less) from the intervention by regressing the outcomes on independent variables such as gender, education, mild/moderate depressive symptoms, the treatment dummy, and the interaction with the treatment dummy and the selected independent variables.

The economic evaluation will be conducted from a societal perspective, thus including the intervention costs (ie, Psyfit), the costs of health care uptake (TIC-P), the participants’ out-of-pocket costs for obtaining health care (TIC-P), and the economic costs due to productivity losses in paid work (PRODISQ). The incremental cost-effectiveness ratio (ICER) will be calculated. Uncertainty in the ICER will be captured using a bootstrap approach, producing a scatter of simulated ICERs over the ICER-plane and by drawing an ICER acceptability curve of the likelihood that Psyfit is more cost-effective for a range of willingness-to-pay (WTP) ceilings.

All analyses will be conducted using two-sided tests and alphas of .05. For this purpose, the most recent version of the SPSS software will be used.

## Discussion

This online trial will examine whether an Internet-based self-help intervention for the enhancement of well-being is effective in terms of clinical outcomes and economic costs. A second objective is to examine whether certain groups benefit more or less from the intervention. The enhancement of well-being on a large scale may contribute to public mental health by resulting in better health, fewer mental disorders, and enhanced quality of life on a population level [11,70].

This study has several *a priori *limitations. First, dropout may occur in either of the two groups. To examine any selectiveness, we will conduct dropout analysis and a telephone survey for examining reasons for dropout. Moreover, we will conduct intention-to-treat analyses in which missing values are replaced by their most likely estimates. Second, we will only use self-report questionnaires, not formal diagnostic instruments, to establish diagnoses. Therefore, we will not know whether participants meet the criteria for a Diagnostic and Statistical Manual of Mental Disorders (DSM-IV) diagnosis so results about prevention of mental disorders will not be available. We opted for self-rating because the intervention should be easily accessible and highly applicable because of its public nature. We don’t want to scare people off by intensive diagnostic procedures. A third limitation of this study concerns the use of questionnaires that are not (yet) validated for online purposes. Psychometric properties of online assessments may differ from their paper-and-pencil counterparts [71]. On the other hand, the CES-D [45] and the MHC-SF [52], which are used in this study, have been proven to be reliable and valid instruments even if used on the Internet. Finally, the open recruitment strategy may attract certain groups, for example, more higher-educated people or more spiritually engaged and higher-motivated people. Therefore, care must be taken in generalizing the results.

Our study also has several strengths. It is likely to add to the existing literature because it is—at least to our knowledge—the first evaluation of an online flexible and multiple-component intervention aimed at the improvement of well-being to include people with mild depressive symptoms. As such, we will be able to draw conclusions about the potential impact of such an intervention on mental health. Although the recruitment procedure could be a weakness as previously mentioned, at the same time it creates the opportunity to strengthen external validity (real world implementation potential) by analyzing which target groups are attracted by the “open access” and positively formulated recruitment strategy.

Online interactive programs may attract large numbers of people, therefore, even small or moderate effect sizes can have an impact on population health. If proven to be effective, Psyfit may be an affordable instrument that can be distributed on a large scale to enhance population well-being.

## Multimedia Appendix 1

CONSORT-EHEALTH checklist (V1.6) [72].

## Abbreviations

CES-D: Center for Epidemiological Studies Depression Scale
CONSORT: Consolidated Standards for Reporting Trials
CSQ-8: Client Satisfaction Questionnaire
DSM-IV: Diagnostic and Statistical Manual of Mental Disorders, Fourth Edition
EM: expectation-maximization
ICER: incremental cost-effectiveness ratio
IP: Internet Protocol
ITT: intention-to-treat
METIGG: Medical Ethics Committee for Mental Health Care
MHC-SF: Mental Health Continuum-Short Form
MOS SF-36: Medical Outcomes Study Short Form (36 items)
PRODISQ: Productivity and Disease Questionnaire
SPSS: Statistical Package for the Social Sciences
TIC-P: Trimbos Institute and Institute of Medical Technology questionnaire for Costs associated with Psychiatric illness
WHO: World Health Organization
WHO-5: WHO-Five Well-being Index
WTP: willingness-to-pay
