# Supplementary material for: Internet-Based Intervention to Promote Mental Fitness in Mildly Depressed Adults: Design of a Randomized Controlled Trial
Source: JMIR Res Protoc. 2012 Apr 26;1(1):e2. doi: 10.2196/resprot.1791 (PMC3626141; doi:10.2196/resprot.1791)
Supplement: Supplementary file 1 [file resprot_v1i1e2_app1.pdf]

CONSORT-EHEALTH Checklist V1.6 Report

Manuscript Number 1791
Date completed 8/25/2011 14:50:29
by Linda Bolier

Internet-based intervention to promote well-being: design of an online pragmatic randomized controlled trial

TITLE

1a-i) Identify the mode of delivery in the title
Yes, "Internet-based intervention"

1a-ii) Non-web-based components or important co-interventions in title
Not mentioned because the e-mailreminding is part of, but not a central element in the intervention.

1a-iii) Primary condition or target group in the title
No, not at first. I added "for adults interested in improving their mental fitness" in the title.

ABSTRACT

1b-i) Key features/functionalities/components of the intervention and comparator in the METHODS section of the ABSTRACT
Yes, though not elaborately.
"Investing in mental well-being is seen as an addition to current mental health service delivery in which the treatment and prevention of mental disorders are core components. It is may be possible for people to enhance well-being by enhancing their mental fitness, in a way that is analogous to improving their physical condition.
Psyfit, an online multi-component self-help intervention was developed with the aim of improving well-being and reducing depressive symptoms."

"a 6-month waiting list (control condition)"

1b-ii) Level of human involvement in the METHODS section of the ABSTRACT
Yes.
"and fully-automated self-help intervention"

1b-iii) Open vs. closed, web-based (self-assessment) vs. face-to-face assessments in the METHODS section of the ABSTRACT
Yes: "Online measurements by self-assessment are made prior to randomization .."

I did not mention the blinding process because it is clear that using a waiting list implies automatically 'unblinded' .

1b-iv) RESULTS section in abstract must contain use data
Yes:
"Primary outcome is well-being. Secondary outcomes are depressive symptoms, general health, vitality and economic costs. Analysis will be conducted in agreement with the intention-to-treat principle."

1b-v) CONCLUSIONS/DISCUSSION in abstract for negative trials
No, because it is a protocol article.

INTRODUCTION

2a-i) Problem and the type of system/solution
Yes, problem analysis:
"The available evidence suggests that promotion of well-being might be a necessary public health strategy in mental health promotion.

"Taking a positive mental health approach, an internet-based self-help intervention ('Psyfit') aimed at the promotion of well-being was developed. Below, we elaborate on the public health rationale for this type of intervention: the definition of well-being, the relevance of the internet as an implementation vehicle and the use of positive psychology interventions as a starting point."

2a-ii) Scientific background, rationale: What is known about the (type of) system
Yes:
"Positive psychology interventions
The positive psychology movement has developed many interventions which focus on flourishing and positive functioning, for example by counting your blessings [27-29], practicing kindness [30,31], setting your personal goals [32-34], expressing gratitude [28,29] and using your personal strengths [28]. A comprehensive meta-analysis of 51 positive psychology interventions demonstrated moderate effect sizes for enhancing well-being and reducing depressive symptoms [35].
However, experimental research on well-being interventions that are offered over the internet is still scarce and results are mixed. In Recently a randomized controlled trial with two single interventions (working with your strengths and problem solving) and a placebo control group showed mixed results [36]. Well-being was improved but there were no significant impacts on mental illness. In another trial [28] the internet was used for the recruitment of participants and the collection of data. The single exercises 'using signature strengths in a new way' and 'recapitulating three good things' enhanced well-being and reduced depressive symptoms, up to three months. Also 'writing and reading a gratitude letter' was effective but only in the short term. However, a more critical look at the interventions reveal that these exercises were not internet-based interventions in the true sense of the word as is shown in the previously cited definition of Ritterband (2003) [21] because the interventions were neither interactive nor personalised. Also, two randomized controlled trials examining the effects of multi-component interventions were conducted [70, 71]. In a workplace setting an intervention called ' Resilience Online' demonstrated no significant effects [70]. In another study using an online version of positive psychotherapy depressive symptoms were significantly reduced, but there was no improvement of subjective well-being [71].

" Current study
The aim of this study is to evaluate the efficacy and cost-effectiveness of Psyfit, an online well-being program [69]. The study will add to existing literature by testing a multiple and flexible internet-based intervention to promote well-being. To our knowledge all the research done so far is limited to single interventions focusing on one well-being exercise at a time [28, 36] and to multiple protocolized interventions [70, 71]. In these interventions mentioned above, people in the intervention group of the research study are allocated to an inflexible intervention, while in practice people would like to choose what they need and feel up to [30,37,38]. It is for this reason that Psyfit offers a choice of different interventions that people can tailor themselves.
The primary objective of this study is to evaluate the effectiveness of the Psyfit intervention in comparison to a waiting list control group. It is hypothesized that the intervention group will demonstrate a significant increase in well-being and a decrease in depressive symptoms at post-test and follow-up compared to the control group. Secondary study objectives are to conduct an economic evaluation and to examine if particular subgroups benefit more or less than others from the intervention."

METHODS

3a) CONSORT
Yes: "It is hypothesized that the intervention group will demonstrate a significant increase in well-being and a decrease in depressive symptoms at post-test and follow-up compared to the control group. Secondary study objectives are to conduct an economic evaluation and to examine if particular subgroups benefit more or less than others from the intervention."

3b-i) Bug fixes, Downtimes, Content Changes
No, there were no major content changes or downtimes during the trial.

4a-i) Computer / Internet literacy
More or less, two of the inclusion criteria:
" 4) have access to a computer and internet; 5) have sufficient knowledge of the Dutch language"

4a-ii) Open vs. closed, web-based vs. face-to-face assessments:
Yes
"Recruitment
Participants will be recruited through banners on internet websites related to mental health and well-being. In addition, advertisements will be placed in newspapers and monthly magazines on health related topics. (..)
The advertisement includes the website address where people can register (www.psyfit.nl). This website contains all the information about the study and a demonstration video of the intervention. When interested in participation people can leave their name and e-mail address. E-mail and IP addresses will be checked for multiple registrations. Following this people will receive an e-mail with additional information about the study and a link to the online informed consent form and online questionnaire."

4a-iii) Information giving during recruitment

Yes:
"The recruitment message for the study is formulated positively (and not with a focus on symptoms and problems): ‘Would you like to increase your mental fitness? Would you like to feel better? Improve your mental fitness and participate in our study of an online self-help program Psyfit’. The analogy is made with physical fitness: 'there are certain lifestyle behaviors you could adopt that make you feel mentally fit'. From preliminary focus group research it was already known that in practice people with minor mental problems, experiencing stress or who just ‘don’t feel good’ will be attracted by the ‘mental fitness’ message [39]. "

Informed consent;
"Following this people will receive an e-mail with additional information about the study and a link to the online informed consent form and online questionnaire.(..)

On returning the informed consent form and completion of the baseline questionnaire, people who meet the inclusion criteria will be randomly allocated to the experimental group."

4b-i) Report if outcomes were (self-)assessed through online questionnaires

Yes: "All measurements are self-report measures and administered through an e-mail which provide a link to the questionnaire on the internet."

4b-ii) Report how institutional affiliations are displayed

No, this is not reported, nor is the impact measured, but is a good suggestion.

5-i) Mention names, credential, affiliations of the developers, sponsors, and owners

Yes "The study is funded by the Dutch Ministry of Health, Welfare and Sport.

Conflicts of interest
Linda Bolier and Merel Haverman are the developers of Psyfit.nl."

5-ii) Describe the history/development process

Partly.
We did do focus group research and usability testing. Only the focus group is briefly mentioned in the paper:
"From preliminary focus group research it was already known that in practice people with minor mental problems, experiencing stress or who just ‘don’t feel good’ will be attracted by the ‘mental fitness’ message."

5-iii) Revisions and updating

No, it was the first version of Psyfit, and only this version was used.

5-iv) Quality assurance methods

No, it was not mentioned in the paper, but all important safety and privacy standards are applicated in Psyfit.

5-v) Ensure replicability by publishing the source code, and/or providing screenshots/screen-capture video, and/or providing flowcharts of the algorithms used

No. This would require another paper! There is no room to do this because Psyfit is a very comprehensive intervention.
Of course, screenshots and demo-inlogs are available for people who are interested in the intervention.

5-vi) Digital preservation

Yes, one of the references:
"(69)www.psyfit.nl. Archived at Webcite January 28 http://www.webcitation.org/650azWEEe"

5-vii) Access

Yes" An e-mail is sent to each participant assigned to Psyfit with a personal username and password. From the moment the participant logs on, a two month free access to the intervention is activated. If a participant doesn’t log on a reminder e-mail is sent with the log-on codes after one week, and if necessary after two weeks and again after three weeks. Participants are allowed to use the program at any time they want during the trial period."

5-viii) Mode of delivery, features/functionalities/components of the intervention and comparator, and the theoretical framework

Yes.
'Intervention group Psyfit
Psyfit is offered as an online and fully-automated self-help intervention without active support from a therapist. Participants tailor their own intervention program to their personal needs and measure their progress by several self-tests. In addition they can exchange their experiences in an online community which can be accessed via Psyfit.

The content of the well-being program Psyfit is based on an extensive literature search [10]. Elements in the intervention originate from positive psychology [28,35], mindfulness [44], cognitive behavioural therapy [45] and problem solving therapy [46].
Psyfit consists of six modules, each containing a four-week program:
1)Mission and goals (living from a deeply felt mission and personal values)
2)Positive feelings (positive thinking and working on your positive affect)
3)Positive relations (connection with other people and your environment)
4)Living in the moment (consciously living and enjoying)
5)Thinking and feeling (change negative thinking patterns, optimistic thinking)
6)Master your life (managing personal energy, stress and problems)

In theory, each of these modules is likely to have impact on well-being. (…) Participants are recommended to choose and finish at least one module during the intervention period. During a four week module participants receive background information on the specific subject, view short films and each week receive one assignment which they are expected to complete during that week. On average this takes 20 to 30 minutes each day.
Participant are free to use all other functionalities offered in Psyfit and can always choose to start a new module. For an overview of functionalities see Table 1.

Table 1 Functionalities in Psyfit

•General self-test to assess individual well-being level before and after 2 months.
•A personal plan in which the participant can reflect on his or her goals, motives and pitfalls.
•A ‘mood meter’ for monitoring changes in the mood of the participant. The outcomes are presented in a graph.
•Automatic e-mail service twice a week with reminders and tips & advice.
•Online Community for sharing experiences and peer-to-peer support.
•Contact form: participants can ask questions and receive feedback from a psychologist via e-mail. If required, the participant is referred to professional care.
•‘My Psyfit’: the participant can download and print out a personal PDF blueprint of the intervention with all the modules, exercises, and progress measurements completed.
•Videos: each module starts with a video showing a Dutch expert explaining the relevance of this particular module.
•Module self-tests: each module starts and ends with a short self-test to see if the particular skill has improved.

The participant can work through the intervention independently (self-help) but can fill in a contact form with a question if necessary. Psyfit could be likened to a tool-box where people can ‘pick and mix’ whatever they like and need."

5-ix) Describe use parameters

Yes.
"In theory, each of these modules is likely to have impact on well-being. (…) Participants are recommended to choose and finish at least one module during the intervention period."

5-x) Clarify the level of human involvement

Yes

'Psyfit is offered as an online and fully-automated self-help intervention without active support from a therapist."

In the functionalities;
"Contact form: participants can ask questions and receive feedback from a psychologist via e-mail. If required, the participant is referred to professional care. Also technical assistance is provided."

5-xi) Report any prompts/reminders used

Yes.

Reminders for log in:
"If a participant doesn't log on a reminder e-mail is sent with the log-on codes after one week, and if necessary after two weeks and again after three weeks. "

Email reminding during the course:
"•Automatic e-mail service twice a week with reminders and tips & advice."

5-xii) Describe any co-interventions (incl. training/support)

No, there are no co-interventions.

6a-i) Online questionnaires: describe if they were validated for online use and apply CHERRIES items to describe how the questionnaires were designed/deployed

Yes, some information is given:

"When applied via internet, the CES-D appears to be a reliable and valid instrument [43]."

And in the discussion:
"A third limitation of this study concerns the use of questionnaires which are not (yet) validated for online purposes. Psychometric properties of online assessments may differ from their paper-and-pencil counterparts [68]. On the other hand, the CES-D [43] and the MHC-SF [49] which are used in this study are proved to be reliable and valid instruments, also if used on the internet."

6a-ii) Describe whether and how "use" (including intensity of use/dosage) was defined/measured/monitored

Yes.
"The web statistics module will systematically track and trace the actions of each participant like the number of log-on times, the time spent on the website and the modules chosen. Hereby adherence to the intervention can be examined."

6a-iii) Describe whether, how, and when qualitative feedback from participants was obtained

Yes.
"For measuring participant satisfaction with the intervention the Dutch version of the Client Satisfaction Questionnaire-short form is used (CSQ-8)"

7a-i) Describe whether and how expected attrition was taken into account when calculating the sample size

Yes.
'Sample size
Depressive symptoms and well-being are are used as a starting point for the power calculation. We aim to be able to show differences between Psyfit and the waiting list control condition with a standardized effect size (Cohen's d) of 0.33 or larger. A standardized effect of 0.33 can be considered as the lower limit of a moderate clinical effect [61] and is based on a meta-analysis of well-being intervention research [35] and a recent randomized controlled trial [36]. To demonstrate this effect and assuming an alpha of 0.05 and a statistical power (1-Beta) of 80% we need 145 participants in each condition, 290 participants in total for the trial."

7b) CONSORT

No, there were no interim analyses

8a) CONSORT

Yes
"Randomization is stratified by gender, education and severity of symptoms (CES-D score 10-15/16-24). A computer-program conducted the allocation using a generated randomization list. Block randomization in blocks of two is performed to ensure equal distribution of participants across conditions. "

8b) CONSORT

Yes: "Randomization is stratified by gender, education and severity of symptoms (CES-D score 10-15/16-24). (..)Block randomization in blocks of two is performed to ensure equal distribution of participants across conditions."

9) CONSORT

No, concealment was not relevant in our trial, there was no interference from the researchers wth the participants.

10) CONSORT

Yes: It was done online by a computer.

11a-i) Specify who was blinded, and who wasn't

There is no blinding in our study. People assigned to the Psyfit group know they are in the intervention group. There are no care providers.

11a-ii) Discuss e.g., whether participants knew which intervention was the "intervention of interest" and which one was the "comparator"

The participants knew, the comparator condition was a waiting list. See 11-a-i.

11b) CONSORT

Not applicable.

12a) CONSORT

Yes.
"To examine differences between the two conditions, we will use multiple regression analyses with the clinical outcomes on continuous measures (MHC-SF, WHO-5, CES-D, Vitality and General health scale from MOS SF-36) as dependent variables and d the intervention dummy as predictor. We will compute standardized effect sizes (Cohen's d). Cohen's d is computed by subtracting the mean post-test score of each condition and dividing the difference by the pooled standard deviation [65]."

12a-i) Imputation techniques to deal with attrition / missing values

Yes.
"We will adhere to the intention-to-treat principle, which means all participants who have been randomized will be included in the analyses. Missing data at t1 and t2 will be imputed using the expectation-maximisation (EM) method, as implemented in SPSS Missing Value Analysis. It imputes missing values by maximum likelihood estimation using the observed data in an iterative process [64]. In online trials drop-out rates are to be expected and sometimes a large amount of missing data has to be imputed.
Therefore, a completers-only analysis and per protocol analysis will be conducted in addition (sensitivity analysis). "

12b) CONSORT

Yes.
"Moderator analyses will be conducted to examine which groups benefit more (or less) from the intervention by regressing the outcomes on independent variables such as gender, education, mild/moderate depressive symptoms, the treatment dummy and the interaction with the treatment dummy and the selected independent variables.

The economic evaluation will be conducted from a societal perspective, thus including the intervention costs (of Psyfit), the costs of health care uptake (TIC-P), the participants' out-of-pocket costs for obtaining health care (TIC-P), and the economic costs due to productivity losses in paid work (PRODISQ). The incremental cost-effectiveness ratio (ICER) will be calculated. Uncertainty in the ICER will be captured using a bootstrap approach, producing a scatter of simulated ICERs over the ICER-plane and by drawing an ICER acceptability curve of the likelihood that Psyfit is more cost-effective for a range of willingness-to-pay (WTP) ceilings."

RESULTS

13a) CONSORT

Not applicable because it is a protocol paper.

13b) CONSORT

Not applicable because it is a protocol paper.

13b-i) Attrition diagram
Not applicable because it is a protocol paper.
14a) CONSORT
Not applicable because it is a protocol paper.
14a-i) Indicate if critical “secular events” fell into the study period
Not applicable because it is a protocol paper.
14b) CONSORT
Not applicable because it is a protocol paper.
15) CONSORT
Not applicable because it is a protocol paper.
15-i) Report demographics associated with digital divide issues
Not applicable because it is a protocol paper.
16-i) Report multiple “denominators” and provide definitions
Not applicable because it is a protocol paper.
16-ii) Primary analysis should be intent-to-treat
Not applicable because it is a protocol paper.
17a) CONSORT
Not applicable because it is a protocol paper.
17a-i) Presentation of process outcomes such as metrics of use and intensity of use
Not applicable because it is a protocol paper.
17b) CONSORT
Not applicable because it is a protocol paper.
18) CONSORT
Not applicable because it is a protocol paper.
18-i) Subgroup analysis of comparing only users
Not applicable because it is a protocol paper.
19) CONSORT
Not applicable because it is a protocol paper.
19-i) Include privacy breaches, technical problems
Not applicable because it is a protocol paper.
19-ii) Include qualitative feedback from participants or observations from staff/researchers
Not applicable because it is a protocol paper.

DISCUSSION

20-i) Typical limitations in ehealth trials
Yes
"This study has several a priori limitations. First, drop-out may occur in either of the two groups. To examine any selectiveness we will conduct drop-out analysis and a telephone survey for examining reasons for drop-out. Moreover, we will conduct intention-to-treat analyses in which missing values are replaced by their most likely estimates. Second, we only use self-report questionnaires and no formal diagnostic instruments to establish a diagnosis. That means we do not know whether participants meet the criteria for a DSM-IV diagnosis and results on the prevention of a mental disorder will not be available. We opted for self-rating because the intervention should be easily accessible and highly applicable because of its public nature. We don't want to scare people off by intensive diagnostic procedures. A third limitation of this study concerns the use of questionnaires which are not (yet) validated for online purposes. Psychometric properties of online assessments may differ from their paper-and-pencil counterparts [68]. On the other hand, the CES-D [43] and the MHC-SF [49] which are used in this study are proved to be reliable and valid instruments, also if used on the internet. Finally, the open recruitment strategy may attract certain groups, for example more higher-educated people than lower-educated people or more spiritually engaged and higher motivated people. Generalizing results to the general population must therefore be carefully thought."
21-i) Generalizability to other populations
Yes
"Although the recruitment procedure could be a weakness as mentioned, at the same time it creates the opportunity to strengthen external validity (real world implementation potential) by analyzing which target groups are attracted by the 'open access' and positively formulated recruitment strategy. Online, interactive programs online may attract large numbers of people and therefore even small or moderate effect sizes can have an impact on population health. When proved to be effective Psyfit may be an affordable instrument to be disseminated on a large scale for enhancing population’s well-being.   "
21-ii) Discuss if there were elements in the RCT that would be different in a routine application setting
No, because when the trial started it was not clear yet what routine implementation would be.
22-i) Restate study questions and summarize the answers suggested by the data, starting with primary outcomes and process outcomes (use)
Not applicable because it is a protocol paper.
22-ii) Highlight unanswered new questions, suggest future research
Not applicable because it is a protocol paper.

Other information

23) CONSORT
Yes
"The study has been registered by the Netherlands Trial Register, part of the Dutch Cochrane Centre (NTR2126)."
24) CONSORT
http://www.trialregister.nl/trialreg/admin/rctview.asp?TC=2126
25) CONSORT
Yes
"The study is funded by the Dutch Ministry of Health, Welfare and Sport."
X26-i) Comment on ethics committee approval
Yes:
"The study protocol, interventions, participant information and informed consent procedure is approved by the Dutch Medical Ethics Committee for Mental Health Care (METIGG), under registration number 9218. "
x26-ii) Outline informed consent procedures
Yes, but not very elaborately:
"Following this people will receive an e-mail with additional information about the study and a link to the online informed consent form and online questionnaire.(..)

On returning the informed consent form and completion of the baseline questionnaire, people who meet the inclusion criteria will be randomly allocated to the experimental group (Psyfit) or the waiting list and are consequently notified by e-mail."
X26-iii) Safety and security procedures
" participants can ask questions and receive feedback from a psychologist via e-mail. If required, the participant is referred to professional care. "
X27-i) State the relation of the study team towards the system being evaluated
Yes
"Linda Bolier and Merel Haverman are the developers of Psyfit.nl."
